# SheepFaceNet: A Speed–Accuracy Balanced Model for Sheep Face Recognition

**DOI:** 10.3390/ani13121930

**Published:** 2023-06-09

**Authors:** Xiaopeng Li, Yichi Zhang, Shuqin Li

**Affiliations:** College of Information Engineering, Northwest A&F University, Xianyang 712100, China

**Keywords:** sheep face recognition, lightweight model, convolutional neural network, speed-accuracy trade-off, reparameterization

## Abstract

**Simple Summary:**

The use of computer vision technology has improved the effectiveness of individual sheep identification, but existing methods face challenges such as large parameter sizes, slow recognition speeds, and difficult deployment. To address this issue, we have made improvements and optimizations based on the Retinaface face recognition model to create a balanced speed-accuracy sheep face recognition model. The optimized model has fewer parameters, simpler computations, faster inference speeds, and higher recognition accuracy, making it well-suited for deployment on resource-constrained edge devices. This research is expected to promote the application of deep learning-based sheep face recognition methods in production.

**Abstract:**

The recognition of sheep faces based on computer vision has improved the efficiency and effectiveness of individual sheep identification, providing technical support for the development of smart farming. However, current recognition models have problems such as large parameter sizes, slow recognition speed, and difficult deployment. Therefore, this paper proposes an efficient and fast basic module called Eblock and uses it to build a lightweight sheep face recognition model called SheepFaceNet, which achieves the best balance between speed and accuracy. SheepFaceNet includes two modules: SheepFaceNetDet for detection and SheepFaceNetRec for recognition. SheepFaceNetDet uses Eblock to construct the backbone network to enhance feature extraction capability and efficiency, designs a bidirectional FPN layer (BiFPN) to enhance geometric location ability, and optimizes the network structure, which affects inference speed, to achieve fast and accurate sheep face detection. SheepFaceNetRec uses Eblock to construct the feature extraction network, uses ECA channel attention to improve the effectiveness of feature extraction, and uses multi-scale feature fusion to achieve fast and accurate sheep face recognition. On our self-built sheep face dataset, SheepFaceNet recognized 387 sheep face images per second with an accuracy rate of 97.75%, achieving an advanced balance between speed and accuracy. This research is expected to further promote the application of deep-learning-based sheep face recognition methods in production.

## 1. Introduction

Sheep play a crucial role in animal husbandry and are essential for agricultural production and the human diet. Traditional sheep individual identification methods usually use artificial marking, whereby a tag or magnetic disk is attached to the sheep’s ear, and identity confirmation is done through handheld readers. However, this method has many problems, such as easy loss, damage, and impersonation of tags [1], and it is difficult for the approach to meet the high load management requirements of large-scale farms [2]. In addition, long-term wearing of tags may also cause physical and psychological harm to sheep [3].

In contrast, sheep face recognition technology has the advantages of contactless, non-invasive, rapid, and accurate characteristics, which can effectively avoid the problems of artificial marking and improve breeding efficiency and production benefits. Deep-learning-based sheep face recognition mainly uses convolutional neural network architecture [4] and training methods to learn features and patterns from a large number of sheep face images for automatic recognition and classification. Similar to the process of facial recognition, sheep face recognition mainly includes two parts: sheep face detection and recognition. Sheep face detection is used to determine whether there is a sheep face in the image, and the size and location of the sheep face in the image, and crops the whole sheep face area from the image for subsequent sheep face recognition. Sheep face recognition extracts features through a convolutional neural network, represents the sheep face as a vector, compares it with the sheep faces stored in a database, finds the one with the highest similarity, and gives the identity information of the sheep. For example, [5,6,7,8,9,10] improved the efficiency and effectiveness of sheep face recognition using neural-network-based methods with high recognition accuracy. However, they use heavyweight neural networks for sheep face detection and recognition, leading to a large number of parameters in the recognition model, complex computations, slow inference speed, and impossible deployment on resource-limited edge devices. To address this issue, [11,12,13] designed lightweight feature extraction networks to reduce the number of model parameters and computational complexity for deployment on edge devices. Although these works effectively reduce the number of model parameters and complexity, related studies [14] show that reducing the number of parameters and computational complexity cannot effectively improve the speed of the model during inference, and the model may not meet the real-time requirements of actual production. Therefore, [15,16,17,18,19,20] designed real-time recognition models, which effectively improve the recognition speed of the model; however, the recognition accuracy needs to be improved. Therefore, we urgently need a recognition model that strikes a good balance between recognition accuracy and recognition speed.

We propose a computationally efficient and fast basic module, Eblock. Eblock uses inexpensive linear operations to complement redundant features and employs reparameterization during inference to reduce model parameters and calculation complexity, accelerating model inference. Based on this, we built a lightweight sheep face recognition model, SheepFaceNet, achieving the best speed–accuracy trade-off. SheepFaceNet consists of a detection module, SheepFaceNetDet, and a recognition module, SheepFaceNetRec. SheepFaceNetDet uses Eblock to enhance feature extraction efficiency and speed, designs a BiFPN layer to enhance model geometric positioning ability, and optimizes the network structure, which affects inference speed, achieving fast and accurate sheep face detection. SheepFaceNetRec uses Eblock to construct a feature extraction network, employs ECA channel attention to improve the effectiveness of feature extraction, and uses multi-scale feature fusion to achieve fast and accurate sheep face recognition. On our self-built sheep face dataset, SheepFaceNet recognized 387 sheep face images per second with a recognition accuracy of 97.75%, achieving the state-of-the-art speed–accuracy trade-off. This research is expected to further promote the application of deep-learning-based sheep face recognition methods in production.

Our contributions are summarized as follows:

(1) Proposing the efficient and fast basic module Eblock;
(2) Proposing the lightweight sheep face recognition model SheepFaceNet;
(3) Achieving the state-of-the-art speed–accuracy trade-off using SheepFaceNet.

## 2. Materials and Methods

### 2.1. Dataset

The sheep face image data used in this paper come from the Yanchi Tan sheep breeding base in Ningxia, China. A total of 95 segments of sheep face videos were obtained by tracking and shooting the sheep faces in different environments such as indoors, outdoors, in different lighting, at different distances, and at different heights. The videos cover 78 dairy goats and 61 Tan sheep, with a resolution of 1920 × 1080 pixels. These videos were then divided into frames using FFmpeg, and the SSIM algorithm [21] was used to delete images with high similarity in order to avoid too much interference in the network training.

**Figure 1:**
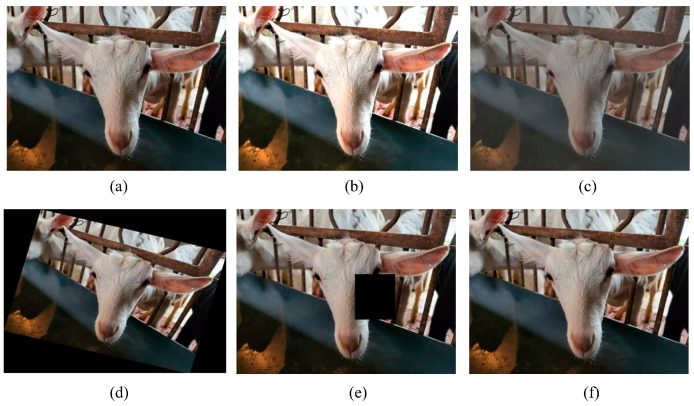
Enhanced results of sheep face images. (**a**) represents the original image, (**b**) represents the image after brightness adjustment, (**c**) represents the image after blurring, (**d**) represents the image after rotation, (**e**) represents the image after occlusion, and (**f**) represents the image after contrast adjustment.

A total of 7328 images were obtained, and these raw datasets were divided into a training set, validation set, and test set in a ratio of 6:3:1. To expand the dataset and improve the model’s generalization ability, the ImageEnhance module in the PIL library of Python was used to augment the original dataset. The specific augmentation methods include adjustment of brightness, contrast, rotation, occlusion, random cropping, etc., and the enhanced examples are shown in Figure 1. After data augmentation, the training set contained a total of 16,812 images.

**Figure 2:**
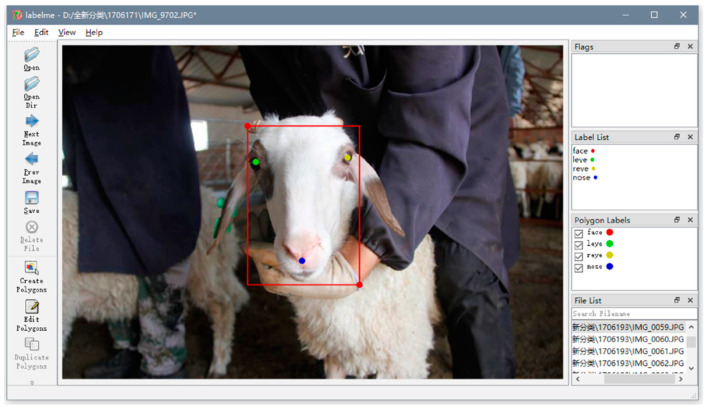
The process of labeling sheep faces.

To more accurately detect sheep faces, the Labelme tool [22] was used in this paper to annotate the sheep faces in the obtained images. When annotating, referring to the production method of the face recognition dataset, the main facial area of the sheep face was selected as the detection box, and key points, such as the sheep’s eyes and nose, were labeled, as shown in Figure 2.

### 2.2. Efficient and Fast Basic Module EBlock

To reduce the number of parameters and computational complexity of the model and improve the inference speed, this paper proposes an efficient and fast basic module called Eblock. Deep convolutional neural networks typically consist of many convolutional layers, which leads to a huge computational cost. Some recent works, such as MobileNet [23] and ShuffleNet [24], have introduced deep convolution or shuffle operations to build efficient convolutional neural networks using smaller convolution kernels, but the remaining 1 × 1 convolution layers still consume a considerable amount of memory and parameters. Assuming the shape of input data X is $X\in R^{c\times h\times w}$, where *c*, *h*, and *w* represent the number of channels, height, and width, respectively, the operation that generates *n* feature maps can be represented as:

(1) Y=X∗F+B

**Figure 3:**
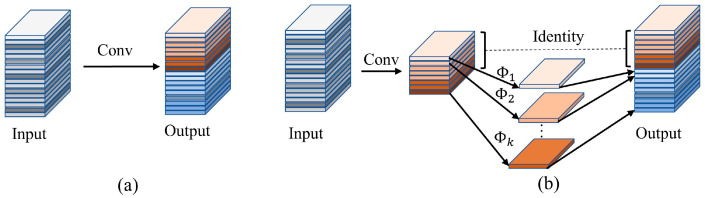
Traditional convolution and Ghost module. (**a**) is the traditional convolutional structure and (**b**) is the Ghost module in [25].

In the equation, ∗ represents the convolution operation, *B* is the bias term, $Y\in R^{h^{\prime }\times w^{\prime }\times n}$ is the output feature map with n channels, and $h^{\prime }$ and $w^{\prime }$ are the height and width, respectively, of the output. $F\in R^{c\times k\times k\times n}$ is the convolution kernel in this layer and *k* × *k* is the size of F. In this convolution process, the calculation method of FLOPs is $n\cdot h^{\prime }\cdot w^{\prime }\cdot c\cdot k\cdot k$. The number of convolution kernels n and the number of channels c are usually very large, resulting in a large quantity of FLOPs, as shown in Figure 3a.

Mainstream convolutional neural networks usually generate redundancy when computing feature maps and these redundancies are necessary for the performance of the network. However, related research [14] has shown that it is not necessary to use a large number of parameters and FLOPs to generate these redundant feature maps one by one. Simple linear transformations can generate redundant feature maps from intrinsic feature maps. The process of generating m intrinsic feature maps $Y^{\prime }\in R^{h^{\prime }\times w^{\prime }\times m}$ is shown in (2), where m is far less than *n*. This process is generated by a primary convolution with convolution kernel parameters identical to those in Equation (1). Here, $F^{\prime }\in R^{c\times k\times k\times m}$ is the convolution kernel used.

(2) $$Y^{\prime }=X*F^{\prime }$$

The process of using inexpensive linear transformations to generate n redundant feature maps from m intrinsic feature maps is shown below. Here, $y_{i}^{\prime }$ is the *i*-th intrinsic feature map in $Y^{\prime }$, and $\Phi_{i,j}$ is the *j*-th linear transformation used to generate the *j*-th feature map $y_{ij}$ in Equation (3). Using Equation (3), we can obtain *n* = m ∗ s feature maps $Y=\left[y_{11,}\;y_{12},\ldots y_{ms}\right]$. Linear transformations are performed on each channel, and their computational cost is much lower than that of ordinary convolutions. The structure is shown in Figure 3b.

(3) $$y_{ij}=\Phi_{i,j}\left(y_{i}^{\prime }\right),\;\forall \;i=1,\ldots ,m,\;j=1,\ldots ,s.$$

**Figure 4:**
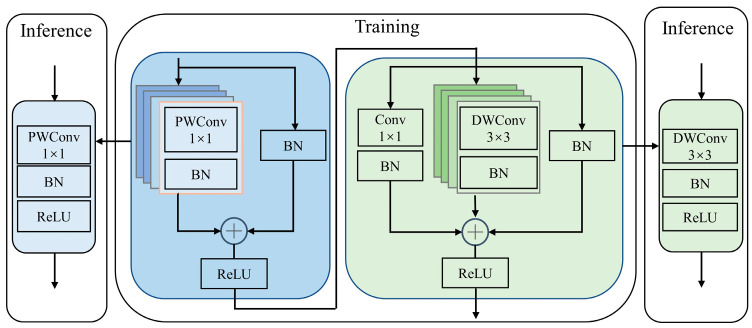
Structure of Eblock.

The above operation can significantly reduce the number of model parameters and computational complexity, but it cannot significantly reduce the inference speed of the model. Therefore, this paper proposes to reparameterize the process described in Equation (3) to improve the inference speed and build an efficient and fast basic module, Eblock. Its structure is shown in Figure 4.

Structural reparameterization technology [26,27] is an effective neural network technology that decouples the training phase and the inference phase. In the training phase, for a given backbone network, reparameterization technology increases the model’s representational power by adding multiple branches or specific layers with various neural network components to the backbone network. In the inference phase, the added branches or layers can be merged into the parameters of the backbone network through some equivalent transformations, which can significantly reduce the number of parameters or computational costs without affecting performance and accelerate inference.

**Figure 5:**
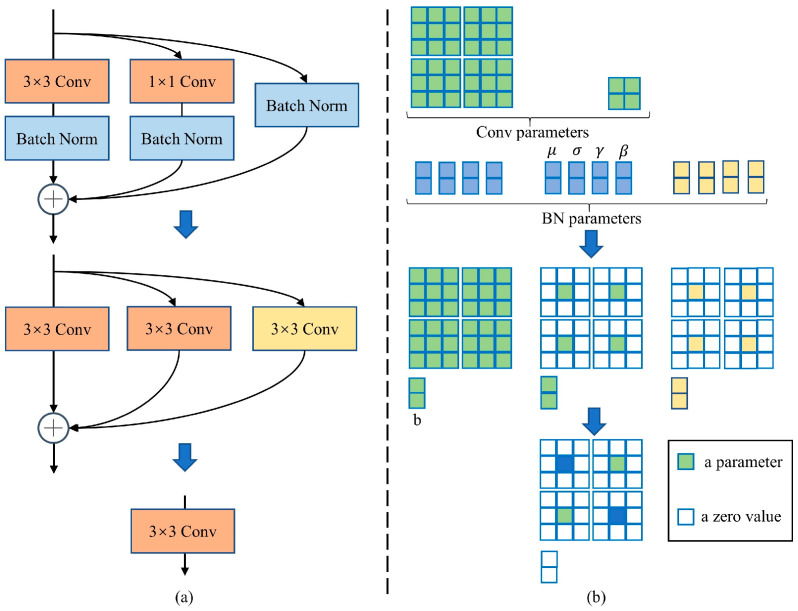
Reparameterization process. (**a**) is the original structure and (**b**) is the reparameterized structure.

The process of reparameterizing the process described in Equation (3) is shown in Figure 5. During training, for the convolution layer with kernel size *K* = {1,3}, input channel $C_{in}$, and output channel $C_{out}$, the weight matrix can be represented as $W^{\prime }\in "{\mathbb{R}}^{C_{out}\times C_{in}\times K\times K}$, and the bias is represented as $B^{\prime }\in {\mathbb{R}}^{D}$. The BatchNorm (BN) layer contains accumulated mean *μ*, variance *σ*, bias *β*, and scaling factor *γ*. Since convolution and BN are linear operations during inference, they can be merged, and the corresponding weight is $\hat{\;W}=W^{\prime }*\frac{\gamma }{\sigma }$ and bias is $\hat{\;B}=\left(B^{\prime }-\mu \right)*\frac{\gamma }{\sigma }+\beta$. For skip connections, BN is merged into the 1 × 1 identity kernel and then zero-padded. After merging BN into each branch, the corresponding weight matrix is $W=\overset{M}{\underset{i}{\sum }}\hat{W_{i}}$ and bias is $B=\overset{M}{\underset{i}{\sum }}\hat{B_{i}}$, where M is the number of network branches. In this way, the model’s parameters and computational costs are greatly reduced, and the inference speed is also accelerated. 

### 2.3. SheepFaceNet

**Figure 6:**
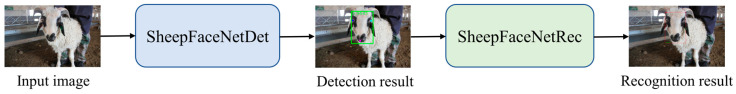
SheepFaceNet sheep face recognition process.

SheepFaceNet is a facial recognition model for sheep that follows the general process of sheep face recognition, which mainly includes two parts: SheepFaceNetDet for sheep face detection and SheepFaceNetRec for recognition, as shown in Figure 6. SheepFaceNetDet uses a sheep face detection dataset to train a sheep face detection model based on SheepFaceNetDet, which extracts the sheep face area from the sheep face image and prepares it for subsequent recognition. SheepFaceNetRec uses the obtained sheep face area for sheep face recognition and outputs sheep face identity information.

### 2.4. Sheep Face Detection Model, SheepFaceNetDet

**Figure 7:**
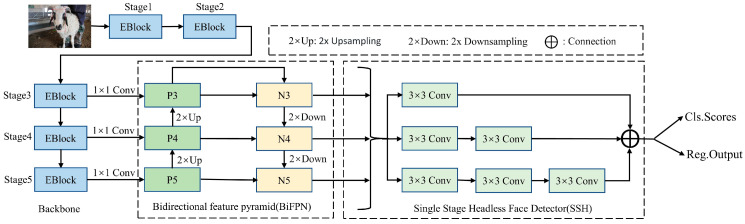
SheepFaceNetDet sheep face detection model structure.

SheepFaceNetDet inherits the structure of RetinaFace [28], a widely used facial detection model, including three parts: feature extraction backbone network, BiFPN module, and SSH feature enhancement module. The feature extraction backbone network performs spatial feature extraction on the input sheep face image and obtains feature maps of different scales in the last three stages. Then, BiFPN is used to fuse detailed information and semantic information, and finally, SSH is used to enhance the receptive field. Its structure is shown in Figure 7.

#### 2.4.1. Feature Extraction Network Based on Eblock

MobilenetV1-0.25 is a lightweight backbone network used by RetinaFace, which uses depthwise separable convolution to reduce the parameter quantity of the model and improve inference speed. It is widely used in object detection tasks. However, its relatively weak feature extraction capability and insufficient perception ability of local structures result in certain impacts on the recognition effect. For example, in the original RetinaFace paper, the AP value of the RetinaFace face detection model with ResNet50 as the feature extraction network is 96.94%, while the AP value of the detection model with MobileNet 0.25 as the main feature extraction network is only 78.2%. Eblock and MobilenetV1’s Inverted Residual Block have two differences. First, Eblock supplements redundant features through inexpensive linear operations to reduce network computations. Specifically, Eblock performs pointwise convolution and depthwise convolution on the input tensor and concatenates the results of pointwise convolution and depthwise convolution on the channel dimension as the final output. This approach is exactly the opposite of the Inverted Residual Block. Secondly, Eblock reparameterizes pointwise convolution and depthwise convolution, effectively improving the model’s inference speed. Through these operations, the feature extraction network constructed by Eblock has higher computational efficiency, better feature extraction performance, and faster inference speed. The structure of the feature extraction network based on EBlock is shown in Table 1. The SheepFaceNetDet detection model receives 640 × 640 sheep face images, reduces the feature map size through downsampling in five stages, increases the model channel number, and takes the feature maps $P_{3}$, $P_{4}$, $P_{5}$ from the last three stages as the output for sheep face detection.

#### 2.4.2. BiFPN

The original RetinaFace face detection model uses FPN for multi-scale feature fusion. However, this top-down feature fusion method gradually fuses the semantic information of deep feature maps to shallow ones, which does not significantly improve the geometric information localization ability of each feature layer. High-level neurons usually have strong responses to the entire object, while low-level neurons are more easily activated by local textures, patterns, etc. Therefore, in the FPN network, a top-down branch is used to propagate high-level semantic features to lower layers so that all feature maps have classification ability. In the FPN network, a proposal is assigned to a specific feature level based on its size. This assigns large proposals to larger feature levels and smaller ones to smaller feature levels. Although this approach is simple, it can cause errors. For example, similar proposals with a difference of only 10 pixels may be assigned to different feature levels, which can affect the recognition results. Therefore, this paper improves the FPN of the original RetinaFace detection model by combining the bidirectional feature fusion structure mentioned in [29]. After the unidirectional top-down feature fusion layer, a bottom-up feature fusion layer is added so that low-level features with precise details and position information can help locate large proposals, enhancing the model’s geometric information localization ability. As shown in Figure 7, the feature maps $N_{3}$, $N_{4}$, and $N_{5}$, which simultaneously have rich semantic and detail information after BiFPN, are input into the SSH for final sheep face detection, further improving the accuracy of sheep face detection.

#### 2.4.3. Time-Consumption Analysis

**Figure 8:**
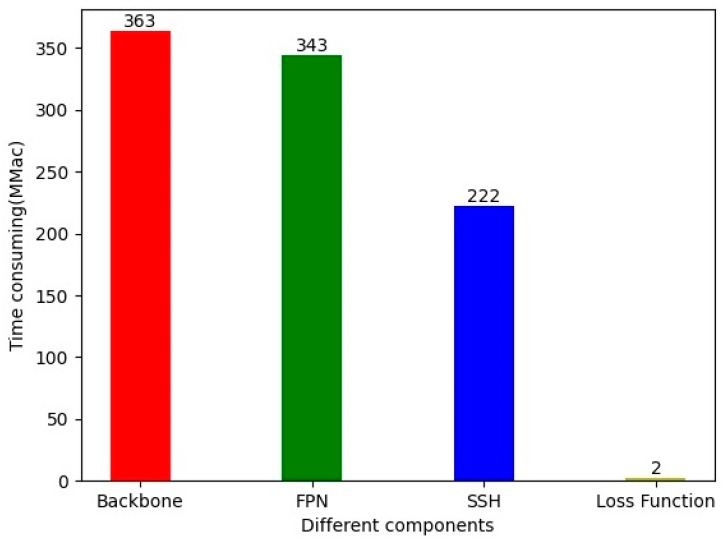
Time-consumption analysis of RetinaFace-Mobilenet0.25.

To further improve the detection speed of the SheepFaceNetDet model, this paper conducts a further time-consumption analysis of all network structures in RetinaFace, using FLOPs as the measurement indicator. As a matter of principle, the actual inference speed of a model is inversely proportional to its FLOPs. Therefore, a larger number of FLOPs means a longer inference time. Although factors such as model parallelism and memory access can also affect inference speed, this paper uses FLOPs to roughly measure the time consumption of each component of the model while keeping these factors as constant as possible. The method used to measure FLOPs in this paper is ptflops.get_model_complexity_info [30]. The experimental results are shown in Figure 8.

It can be seen that the feature extraction network has the highest time consumption, accounting for 39.00% of the total time consumption of the original RetinaFace model, while FPN and SSH [31] account for 36.80% and 23.87%, respectively, and the loss function only accounts for 0.33%. Further analysis found that the feature fusion operation in FPN took the longest time; for example, when fusing the 80 × 80 feature maps with the 40 × 40 feature maps, the time consumption reached 237.16 Mmac, accounting for 24.43% of the total time consumption. The reason for the high time consumption of SSH is similar. Since the feature fusion operation is implemented by regular convolution, in order to further improve the detection speed of the model, this paper replaces all regular convolutions in FPN and SSH with depthwise separable convolutions.

### 2.5. Sheep Face Recognition Method, SheepFaceNetRec

**Figure 9:**
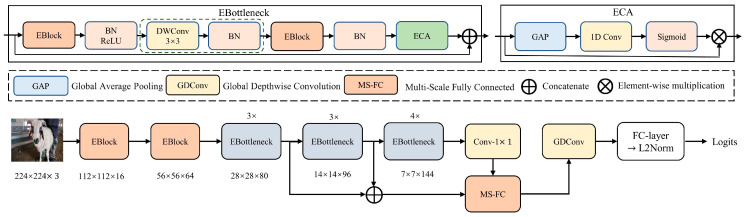
SheepFaceNetRec sheep face recognition model structure.

To address the low recognition accuracy and slow inference speed of the RetinaFace-MobilenetV1-0.25 model, this paper constructed a lightweight and high-accuracy sheep face recognition model using the Eblock as the basis for the feature extraction network, proposing the SheepFaceNetRec lightweight sheep face detection model. The overall structure of SheepFaceNetRec is shown in Figure 9. SheepFaceNetRec uses two Eblocks and ten Ebottlenecks to extract features of different scales. The Eblock is an efficient and fast basic module proposed in this paper, which is used to build the backbone network of the sheep face recognition model to improve the efficiency and effectiveness of feature extraction. The Ebottleneck is used to reduce the number of parameters and computations of the model while maintaining the accuracy of sheep face recognition. The feature maps of sizes 28 × 28 × 80, 14 × 14 × 96, 7 × 7 × 144, and 7 × 7 × 128 are sent to the MS-FC layer through concatenation to provide the model with different receptive fields for recognizing sheep faces of different sizes. The output of the MS-FC layer is then input into the GDConv layer [32] to give different attention to different positions of the feature map. The feature map is flattened into a 128-dimension representation of the sheep face through the FC layer, and the L2 Norm is used to map the sheep face features onto a unit hypersphere to avoid the influence of features in different scales on computational efficiency.

In this paper, the Ebottleneck is proposed by mimicking the structure of the residual block of ResNet to reduce the number of parameters and computations of the model while maintaining recognition accuracy. EBottleneck consists mainly of two EBlocks and one ECA module. The first EBlock serves as an expansion layer to increase the number of channels, while the second EBlock reduces the number of channels to match the residual connection. Residual connections are added between the inputs and outputs of the two EBlocks. After each layer, a BN layer and a ReLU activation function are used. The second EBlock does not use the ReLU activation function. A deep convolutional layer with a stride of 2 is inserted between the two EBlocks. When the stride = 1, the part enclosed by the green dotted line box in EBottleneck does not exist. It should be noted that the BN used here is merged into the corresponding convolution layer during inference.

ECA [33] is an efficient channel attention module that effectively avoids the influence of dimensionality reduction on channel attention learning by using a non-dimensionality reduction local inter-channel interaction strategy. This module improves the performance of the model with a small increase in the number of parameters. The structure of ECA is shown in Figure 8. It first calculates the average value of each dimension feature channel through global average pooling to obtain a vector with a dimension equal to the number of channels. Then, an MLP is used to process the channel vector to obtain a vector representing the weights of each channel. Finally, an accumulation operation is performed.

During the sheep face recognition process, the size of sheep faces in the images varies, thus requiring different receptive fields. This paper borrows from [34] and selects four different scales of feature maps, namely 28 × 28 × 80, 14 × 14 × 96, 7 × 7 × 144, and 7 × 7 × 128, to cover sheep faces of different scales. Meanwhile, these feature maps are relatively small, with low computational complexity and rich semantic information. In this paper, the 28 × 28 feature map is subjected to 4 × 4 average pooling to obtain a 7 × 7 feature map. The 14 × 14 feature map is subjected to 2 × 2 average pooling to obtain a 7 × 7 feature map. Finally, these feature maps with consistent sizes but different channel numbers are connected to form a 7 × 7 × 448 feature map.

## 3. Results

### 3.1. Experimental Environment and Evaluation Index

#### 3.1.1. Experimental Environment

The experimental environment configuration of this paper is shown in Table 2. The pre-training idea in transfer learning is adopted, and the feature extraction network is pre-trained based on the publicly available dataset WIDER FACE [35], which shortens the model training time. After multiple rounds of parameter adjustment, this paper selected parameters with better experimental results. A 640 × 640 × 3 image size is used as the input for the detection model, with a BatchSize of 64 and an SGD optimizer with a momentum of 0.927. The sheep face detection model uses the Complete Intersection Over Union (CIOU) loss function, with an initial learning rate of $1\times 10^{-3}$, a weight decay regularization coefficient of $1\times 10^{-5}$, and a learning rate decay of 10 for model iteration. The sheep face recognition model uses the Arcface loss function, with an initial learning rate of $1\times 10^{-3}$, and the learning rate is adjusted using the cosine annealing algorithm. Dropout is set at 0.6, and 100 epochs are trained.

#### 3.1.2. Evaluation Index

This paper selects the following evaluation metrics: accuracy, precision, recall, average precision (AP), FLOPs, parameters, FPS, and latency. Their calculation methods are shown in Equations (4)–(8), where TP represents true positives, FP represents false positives, TN represents true negatives, and FN represents false negatives. Accuracy refers to the ratio of correctly identified samples to the total number of samples. Precision refers to the ratio of correctly predicted positive samples to the total number of predicted positive samples. Recall refers to the ratio of correctly predicted positive samples to the total number of true positive samples. AP represents the area enclosed by the precision–recall (P-R) curve plotted with recall on the *x*-axis and precision on the *y*-axis, which comprehensively considers the precision and recall indicators of the classifier. FLOPs are commonly used to measure the computational complexity of the model. The smaller the FLOPs value, the lower the model’s computational complexity, and the less time it takes to process. Here, *h*, *w*, and $C_{in}$ represent the height, width, and channel of the input feature map, respectively, while $C_{out}$ represents the channel of the output feature map, and *K* represents the width of the convolution kernel. Parameters are commonly used to calculate the model’s parameter size and model scale, representing the memory size required by the model. Generally, when the number of model parameters is large, the inference time required by the model will be longer. In this paper, FPS is used to measure the speed of the sheep face detector, while latency is used to measure the speed of the sheep face recognition model.

(4) $$Accuracy=\left(TP+TN\right)/\left(TP+TN+FP+FN\right)$$

(5) $$\;\;Precision=TP/\left(TP+FP\right)$$

(6) $$\;Recall=TP/\left(TP+FN\right)$$

(7) $$AP=\int_{0}^{1}P(r)dr$$

(8) $$FLOPs=2hw\times \left(C_{in}\times K^{2}+1\right)\times C_{out}$$

### 3.2. Sheep Face Detection Results

#### 3.2.1. Comparison of Different Sheep Face Detection Models

In order to verify the performance of the SheepFaceNetDet sheep face detection model proposed in this paper, this section compares it with mainstream facial detection models, including Retinaface-Mobilenet0.25, Retinaface-ResNet50, YOLOv5s, and CenterFace [36]. The experimental results are shown in Table 3. It can be seen that the AP of SheepFaceNetDet is 96.36%, slightly lower than the best-performing heavyweight Retinaface-ResNet50, which has an AP of 97.51%. Among all models, SheepFaceNetDet has the least number of parameters, the fastest detection speed, and the lowest computational complexity, achieving the best speed–accuracy trade-off. Compared with the original Retinaface-Mobilenet0.25 model, the SheepFaceNetDet model reduced the number of parameters by 60% and FLOPs by 65%. Meanwhile, the AP increased by 2.77%, and the detection speed was faster, indicating that the improvement of Retinaface-Mobilenet0.25 proposed in this paper is effective. Compared with Retinaface-ResNet50, although the AP is slightly lower, by 1.15%, the number of parameters of Retinaface-ResNet50 is 105 times that of SheepFaceNetDet, and the FLOPs is 60 times that of SheepFaceNetDet. The detection speed is only 34% of the proposed model in this paper. This means that SheepFaceNetDet is not only faster but also has better detection performance. Compared with YOLOv5s and CenterFace, SheepFaceNetDet also shows a better speed–accuracy trade-off.

#### 3.2.2. Ablation Experiment

In order to investigate the effectiveness of the different improvement measures proposed in this paper for RetinaFace, ablation experiments were conducted, and the results are shown in Table 4. “Eblock” represents using Eblock to construct the feature extraction network instead of Mobilenet0.25, “BiFPN” represents using bidirectional FPN, and “DWConv” represents using depthwise separable convolution to replace the ordinary convolution in BiFPN and SSH. It can be seen that due to the weak feature extraction ability of Retinaface-Mobilenet0.25, the detection accuracy is poor, with an AP of only 93.59%. After replacing it with Eblock to construct the feature extraction network, the model’s AP increased by 2.29%, and the detection speed slightly improved, while the changes in parameter count and FLOPs were not significant, which proves the effectiveness of the Eblock design. When replacing the original unidirectional FPN with BiFPN, AP increased by 1.53%, but the detection speed slowed down, and the parameter count and FLOPs slightly increased, indicating that BiFPN can indeed improve detection performance but at the cost of the extra computational burden. When the above two improvements were combined, AP further increased to 96.97%, and the detection speed slowed down slightly. When replacing the convolutions in BiFPN and SSH with depthwise separable convolution, although AP slightly decreased, parameter count and FLOPs significantly reduced, and the detection speed became noticeably faster. Through the above analysis, it can be concluded that the improvements proposed in this paper for Retinaface-Mobilenet0.25 are necessary and effective.

#### 3.2.3. Sheep Face Detection Results

**Figure 10:**
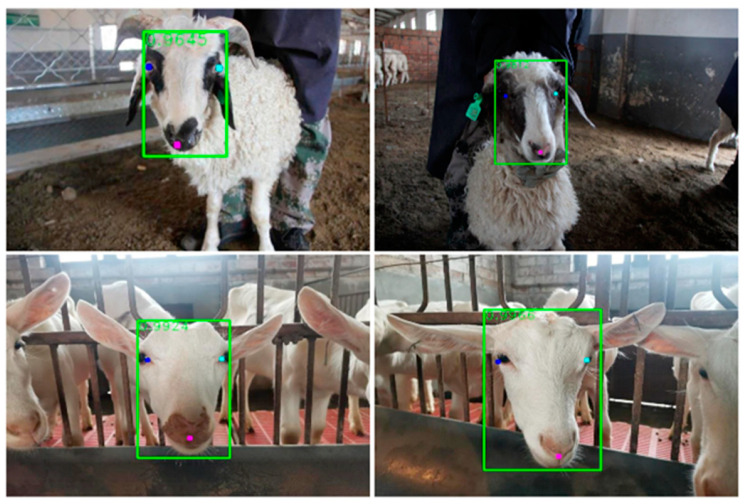
SheepFaceNetDet sheep face detection results.

The detection results of SheepFaceNetDet are shown in Figure 10, which indicates that SheepFaceNetDet can accurately detect the sheep faces in the image and is not affected by the detection environment, demonstrating its excellent performance.

### 3.3. Sheep Face Recognition

#### 3.3.1. Comparison of Recognition Effect with Other Models

To verify the effectiveness of SheepFaceNetRec, this paper compares it with the current state-of-the-art methods ResNet18, MobileNetv1, MobileNetv2 [37], MobileNetv3 [38], EfficientNet-B0 [39], GhostNet, and MobileViT [40]. The experimental results are shown in Table 5. SheepFaceNetRec achieved the best recognition accuracy, similar to that of Resnet18 and Inception-Resnetv2, but with the smallest number of parameters and FLOPs and the lowest latency, demonstrating a good balance between speed and accuracy. Compared with other models, SheepFaceNetRec has different degrees of advantages. For example, compared with the recently proposed lightweight MobileViT-XXS, SheepFaceNetRec’s recognition accuracy is 4.23% higher, and its number of parameters and FLOPs are only 47% and 38% of MobileViT-XXS, respectively, while its latency is only 36%. The effectiveness of SheepFaceNetRec proposed in this paper is evident.

#### 3.3.2. Ablation Experiment

To verify the effectiveness of each component of SheepFaceNetRec, ablation experiments were conducted by separating the two structures in the SheepFaceNetRec structure. The experimental results are shown in Table 6. When nothing is used, SheepFaceNetRec is a relatively lightweight convolutional neural network, with parameters, latency, and recognition accuracy of 0.58 MB, 2.42 ms, and 92.00%, respectively. The recognition accuracy is relatively low. When using ECA to optimize channel feature extraction, the model’s parameters, latency, and recognition accuracy are 0.59 MB, 2.48 ms, and 93.10%, respectively. It can be seen that parameters and latency did not change significantly, but the recognition accuracy increased by 1.1%, demonstrating the effectiveness of ECA. When using multi-scale features for sheep face recognition, the model’s parameters, FLOPs, and recognition accuracy are 0.59 MB, 2.55 ms, and 96.20%, respectively. Compared with the initial model, parameters and latency did not change significantly, but the recognition accuracy increased by 4.2%, and the recognition effect was greatly improved, indicating that MS-FC can indeed improve the model’s recognition effect. When both ECA and MS-FC are used, parameters and latency still do not change much, but the recognition accuracy further improves, eventually reaching 97.75%. Ablation experiments show that each part of the model is necessary and effective.

#### 3.3.3. The Experimental Results of Sheep Face Recognition Are Shown

**Figure 11:**
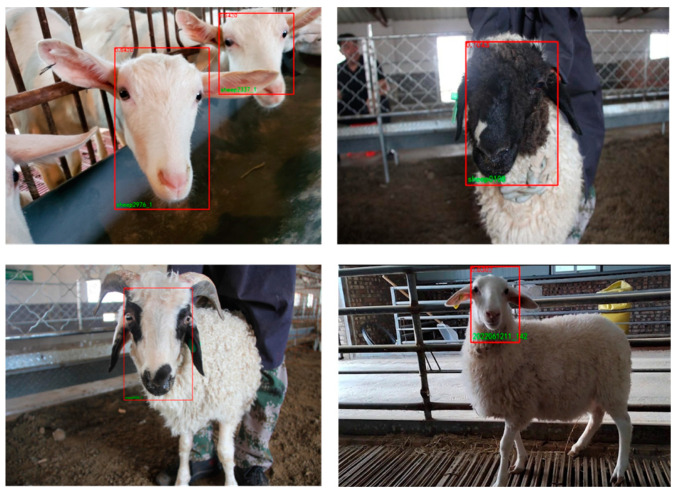
SheepFaceNetRec sheep face recognition results.

Figure 11 shows the results of SheepFaceNetRec sheep face recognition. From the figure, it can be seen that the SheepFaceNetRec sheep face recognition model proposed in this paper can accurately identify the sheep of each color, whether in strong or weak light, and is effective for recognition. This indicates that the SheepFaceNetRec sheep face recognition model proposed in this paper can effectively complete sheep face recognition tasks under most regular conditions.

## 4. Discussion

This paper proposes a lightweight sheep face recognition model, SheepFaceNet, which greatly reduces the number of parameters and computational complexity of the recognition model, improves the inference speed of the model, and has high recognition accuracy, achieving the best speed–accuracy trade-off. It solves the problems faced by sheep face recognition models, such as large parameter sizes, slow recognition speed, and difficult deployment. This paper proposes a computationally efficient and fast basic module, Eblock, and uses Eblock to construct a feature extraction network, which improves the efficiency and effectiveness of the model’s feature extraction and solves the problem that the number of model parameters and computational complexity cannot be reduced to the same extent as the inference speed. To improve the accuracy of the model’s recognition detection and recognition, a bidirectional FPN layer is designed to enhance the model’s geometric localization ability and combined with ECA channel attention and multi-scale feature fusion for sheep face recognition. Comparative experiments with other models show that SheepFaceNet has a fast inference speed and high recognition accuracy. Ablation experiments show that each component of the designed model is effective and necessary. The results of detection and recognition show that SheepFaceNet has great application potential. On a self-built sheep face dataset, SheepFaceNet can recognize 387 sheep face images per second with a recognition accuracy of 97.75%, achieving the best speed–accuracy trade-off. Compared to [5,6,7,8,9,10], SheepFaceNet has a similar recognition accuracy, but its model parameter size, computational complexity, and inference time delay are much lower than those of traditional models. While existing heavyweight backbone networks can achieve good recognition accuracy, their limitations in parameter size, computational complexity, and inference time delay prevent them from being deployed on edge devices. This study designed an efficient and fast basic module Eblock and used it as the basis for designing a lightweight backbone network. Through a series of improvements and optimizations, SheepFaceNet not only achieves high recognition accuracy but also real-time recognition speed. Previous studies [11,12,13] effectively reduced model parameter size and computational complexity by using or improving lightweight recognition models, but their recognition accuracy is lower than that of SheepFaceNet. While MobilenetV2 can reduce model parameters and computational complexity, the use of depthwise separable convolution and pointwise convolution reduces model capacity, thus affecting recognition accuracy. The basic module Eblock proposed in this paper uses inexpensive linear transformations to extract features and reduce feature extraction costs without affecting model capacity. Moreover, previous models do not effectively reduce model inference delays. However, SheepFaceNet’s Eblock improves inference speeds through reparameterization and optimizes time-consuming components of the model, allowing SheepFaceNet to maintain high recognition accuracy while meeting real-time requirements. Previous models [15,16,17] have also implemented sheep face recognition but with low recognition accuracy. For example, [15] has a recognition accuracy of only 91%, which is much lower than SheepFaceNet’s 97.75%. SheepFaceNet strikes a balance between recognition accuracy and speed. It should be noted that the above comparisons are based on results reported by relevant works, and the comparison is only for reference due to the use of different datasets. However, it is enough to show SheepFaceNet’s advantages in the speed–accuracy trade-off. The parameter size and computational complexity of SheepFaceNet are already very low, which meets the requirements for deployment on edge devices. The recognition accuracy of SheepFaceNet is 97.75%, which needs to be further improved for better application in actual production. The above recognition results are based on the dataset constructed in this paper, and we need to further test SheepFaceNet’s performance in actual production. SheepFaceNet accelerates the deployment of deep-learning-based sheep face recognition models on edge devices, promoting the application of sheep face recognition in actual production.

## 5. Conclusions

The lightweight sheep face recognition model proposed in this paper, SheepFaceNet, achieves a balance between speed and accuracy with 0.60 MB of parameters and 0.09 G FLOPs. It can recognize 387 sheep face images per second on our self-built sheep face dataset with an accuracy of 97.75%, surpassing many advanced recognition models. However, the above results are experimental results from our self-built dataset and equipment, and the generalization needs further verification. Moreover, the structure and transformation of the reparameterization in SheepFaceNet need to be redesigned, which is more complicated than ordinary neural networks. The balance between speed and accuracy is essential for the deployment of sheep face recognition models in practice. In the future, we will verify the generalization of SheepFaceNet on other datasets and optimize its speed–accuracy balance to make it more suitable for deployment on edge devices and better serve sheep production.

## Figures and Tables

**Table 1:** Feature extraction network structure of SheepFaceNetDet.

Input	Operation	Input Channel	Output Channel	Feature Map	Stage
640 × 640 × 3	EBlock	8	16	N	0
320 × 320 × 16	EBlock	16	32	N	1
160 × 160 × 32	EBlock	32	64	N	2
80 × 80 × 64	EBlock	64	128	Y	3
40 × 40 × 128	EBlock	128	128	N	4
40 × 40 × 128	EBlock	128	256	Y	4
20 × 20 × 256	EBlock	256	256	N	5
20 × 20 × 256	EBlock	256	256	N	5
20 × 20 × 256	EBlock	256	256	Y	5

**Table 2:** Experimental environment configuration.

Experimental Environment	Configuration Parameters
GPU	NVIDIA GeForce RTX3090
CPU	Intel(R) Core(TM)I7-7700K
Operating system	Ubuntu16.04
Deep Learning Framework	Pytorch 1.10
Programming languages	Python 3.8
GPU Acceleration Library	CUDA11.6, CUDNN 8.3.2

**Table 3:** Comparison of different detection models.

Model	AP (%)	Parameters (MB)	FPS (fps)	FLOPs (G)
Retinaface-Mobilenet0.25	93.59	0.42	75.13	1.29
Retinaface-ResNet50	97.51	27.29	31.52	50.75
YOLOv5s	94.20	7.27	37.82	8.19
CenterFace	92.64	0.38	65.54	1.56
SheepFaceNetDet	96.36	0.25	91.43	0.84

**Table 4:** Results of ablation experiments.

Eblock	BiFPN	DWConv	AP (%)	Parameters (MB)	FPS (fps)	FLOPs (G)
			93.59	0.42	75.13	1.02
√			95.88	0.41	81.75	1.29
	√		95.12	0.57	63.87	1.24
√	√		96.97	0.58	72.11	1.58
√	√	√	96.36	0.25	91.43	0.84

**Table 5:** Comparison results of different models.

Model	Accuracy (%)	Parameters (MB)	Latency (ms)	FLOPs (G)
Resnet18	97.88	11.69	11.30	1.82
MobilenetV2	94.20	3.50	4.27	0.40
MobilenetV3	96.10	2.54	5.33	0.22
EfficientNet-B0	97.78	5.29	6.98	0.38
Inception-Resetv2	97.91	55.84	28.31	6.67
MobileViT-XXS	93.52	1.27	7.01	0.25
MobileViT-XS	95.61	2.32	7.30	0.70
MobileViT-S	95.77	5.58	7.70	1.42
SheepFaceNetRec	97.75	0.60	2.58	0.09

**Table 6:** Results of ablation experiments.

ECA	MS-FC	Parameters (MB)	Latency (ms)	Accuracy (%)
		0.587	2.42	92.00
√		0.596	2.48	93.10
	√	0.593	2.55	96.20
√	√	0.602	2.58	97.75

## Data Availability

The data presented in this study are available upon request from the corresponding author. The data are not publicly available due to the privacy policy of the authors’ institution.
